# sTREM-1 is a specific biomarker of TREM-1 pathway activation

**DOI:** 10.1038/s41423-021-00733-5

**Published:** 2021-07-19

**Authors:** Lucie Jolly, Kévin Carrasco, Margarita Salcedo-Magguilli, Jean-Jacques Garaud, Simon Lambden, Tom van der Poll, Alexandre Mebazaa, Pierre-François Laterre, Sebastien Gibot, Amir Boufenzer, Marc Derive

**Affiliations:** 1 Inotrem, Vandœuvre-lès-Nancy, France; 2 Division of Infectious Diseases, Amsterdam University Medical Centers, University of Amsterdam, Amsterdam, The Netherlands; 3 Intensive Care Unit, Saint Louis-Lariboisière - Fernand Widal University Hospital, Assistance Publique Hôpitaux de Paris, and Université de Paris, Inserm MASCOT, Paris, France; 4 Intensive Care Unit, Université Catholique de Louvain, Louvain-la-Neuve, Belgium; 5 Intensive Care Unit, CHRU de Nancy, and Université de Lorraine, Nancy, France

**Keywords:** Prognostic markers, Biomarkers

TREM-1 (triggering receptor expressed on myeloid cells-1) is a transmembrane receptor expressed by innate immune cells, including endothelial cells and platelets. TREM-1 is a crucial mediator of septic shock that acts by synergizing with Toll-like receptors (TLRs) to amplify the inflammatory responses to pathogens, thus promoting sepsis-induced immune dysregulation and organ dysfunction [1–3].

In addition to its expression in a cell membrane-bound form, TREM-1 is released as a soluble factor (sTREM-1). sTREM-1 has been extensively investigated and seems to be a reliable biomarker of disease severity and outcome, particularly in septic shock [4]. A phase 2 clinical trial including 450 septic shock patients is ongoing to investigate the efficacy of nangibotide, a TREM-1 inhibitor, and a patient selection approach based on baseline sTREM-1 levels (NCT04055909). Here, we aimed to decipher the molecular mechanisms and regulation of TREM-1 expression and sTREM-1 release with the objective of establishing a rationale for the use of sTREM-1 as a marker of TREM-1 pathway activation and as a companion diagnostic marker for therapeutic approaches targeting TREM-1. See supplementary material 1 for the Methods.

Previous reports have suggested proteolytic cleavage of the anchored TREM-1 protein by a matrix metalloproteinase (MMP) as a mechanism of sTREM-1 release [5, 6]. We previously discovered that TREM-1 dimerization is a key step in allowing its activation and the transduction of intracellular signals [7]. This dimerization is induced by mobilization of the receptor at the membrane following TLR activation. We were able to reproduce these findings with the human myelomonocytic cell line U937. Supplementation with vit-D in the medium induced upregulation and diffuse expression of TREM-1 at the membrane (Fig. 1A and B), and stimulation with lipopolysaccharide (LPS) induced TREM-1 clustering at the membrane (Fig. 1B). The addition of activated MMP9 was able to induce sTREM-1 release into the supernatant only under LPS-stimulated conditions (Fig. 1C), confirming that TREM-1 dimerization is essential for the proteolytic cleavage of membrane TREM-1. Activation of TREM-1 induces rapid phosphorylation of Syk (Spleen tyrosine kinase), followed by activation of the TREM-1 pathway, ultimately leading to cytokine release. Incubation of U937-vitD cells with increasing concentrations of the PGN-PGLYRP1 complex (Peptidoglycan - Peptidoglycan Recognition Protein 1), a ligand of TREM-1, induced dose-dependent phosphorylation of Syk and release of IL-6 (Interleukine 6). This treatment also induced dose-dependent release of sTREM-1 into the supernatant (Fig. 1D). These results suggest that sTREM-1 release depends on the activation and dimerization of the receptor and is a marker of TREM-1 receptor activation.

**Fig. 1:**
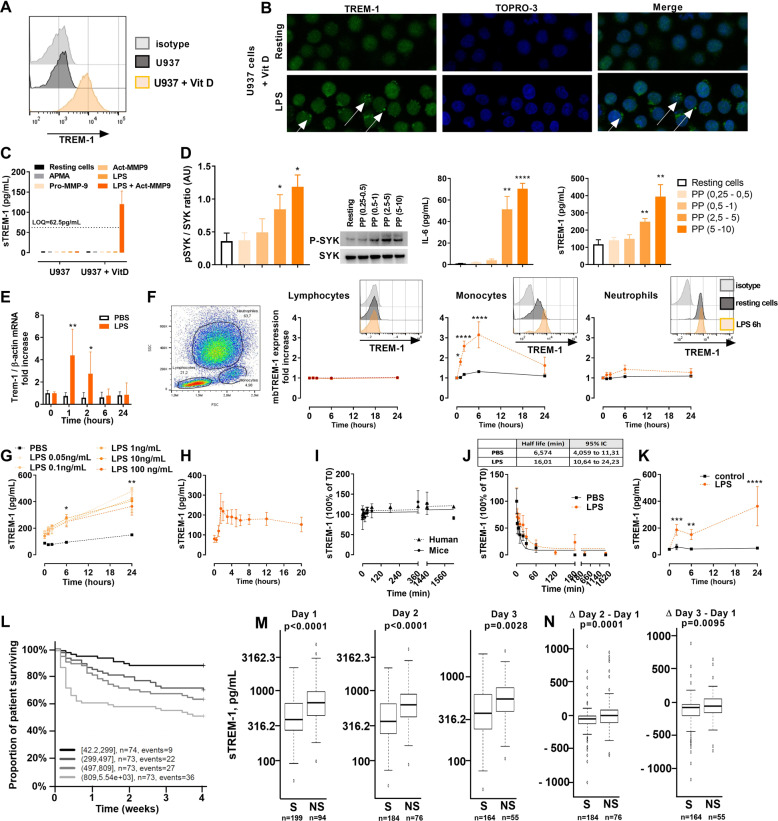
**A**, **B** TREM-1 expression determined by flow cytometric and confocal microscopy analyses of U937 cells in the presence of 100 nM 1,25-dihydroxyvitamin D3 (vitD) or 1 µg/mL lipopolysaccharide as indicated. Nuclei were stained with TO-PRO-3. White arrows: TREM-1 clusters. **C** sTREM-1 concentration in supernatants of U937 cells determined by ELISA. Data are expressed as the mean ± SD, *n* = 3 experiments. LOQ: limit of quantification. **D** U937-vitD cells were stimulated with increasing concentrations of PP (PGLYRP1-PGN complex). Left panel: Western blot analysis of U937-vitD cell lysates after 2 min of stimulation. Graphs show the quantification of the pSyk/Syk ratio (AU arbitrary units). Middle and right panels: IL-6 and sTREM-1 concentrations in supernatants after 24 h of stimulation. Data are expressed as the mean ± SD, *n* = 3 experiments. *p < 0.05, **p < 0.01, ****p < 0.0001 (two-tailed Student’s *t*-test). **E**, **F** TREM-1 expression in blood leucocytes evaluated by flow cytometry and qRT-PCR. Data are expressed as the mean ± SD of seven different donors. *p < 0.05, **p < 0.01, ***p < 0.001, ****p < 0.0001 (two-way ANOVA). **G**–**K** Plasma sTREM-1 concentration determined by ELISA following **G** ex vivo stimulation of human whole blood with LPS (*n* = 5), **H** in vivo administration of LPS to heathy volunteers (2 ng/kg, *n* = 5), **I** ex vivo addition of 2000 pg/mL recombinant TREM-1 protein to mouse and human whole blood (*n* = 3), **J** IV injection of recombinant TREM-1 into TREM-1^−/−^ mice (*n* = 8), and **K** following  intraperitoneal administration of 25 mg/kg LPS to mice (*n* = 10). Data are expressed as the mean ± SD. *p < 0.05, **p < 0.01, ***p < 0.001, ****p < 0.0001 (two-way ANOVA). **L** Kaplan–Meier curves of 28-day mortality according to sTREM-1 quartiles. **M** Plasma sTREM-1 concentration in septic shock patients in survivors and nonsurvivors on day 1 (*n* = 293), day 2 (*n* = 260), and day 3 (*n* = 219). **N** delta-sTREM-1 from day 1 to day 2, and day 1 to day 3

To better understand the kinetics of TREM-1 expression and sTREM-1 release, we stimulated human whole blood with LPS and quantified the mRNA expression of TREM-1; its membrane expression on lymphocytes, monocytes and neutrophils; and the plasma levels of sTREM-1 over time (Fig. 1E–G). We observed a time-dependent induction of TREM-1 expression, starting with an increase in the TREM-1 mRNA level in leucocytes at 1 and 2 h, followed by an increase in TREM-1 membrane expression on monocytes from 2 to 6 hours and a progressive accumulation of sTREM-1 up to 24 h. We also confirmed findings reported in Carrasco et al. [7] showing that TREM-1 is differentially expressed and regulated on monocytes and neutrophils (Fig. 1F). TREM-1 is weakly expressed on monocytes in resting conditions and transiently upregulated following LPS stimulation. The decrease in TREM-1 membrane expression from 6 to 24 h might be due to proteolytic cleavage and release of sTREM-1, which may participate in the progressive increase in sTREM-1 in blood (Fig. 1G). In neutrophils, TREM-1 is highly expressed in resting conditions and not upregulated upon LPS administration. Nevertheless, we previously showed that despite no apparent change in TREM-1 at the membrane upon stimulation, there is dynamic turnover of neutrophil membrane TREM-1, suggesting that neutrophils also contribute to the release of sTREM-1 via cleavage of the membrane-bound receptor [7]. Interestingly, the concentration of plasma sTREM-1 was independent of the concentration of LPS used (from 0.05 to 100 ng/mL), and all doses of LPS induced a progressive increase in sTREM-1 over 24 hours (Fig. 1G). Altogether, these results confirm that TREM-1 is upregulated and sTREM-1 is released in whole blood following LPS stimulation.

A low dose of LPS (2 ng/kg) in humans induced a transient  increase in sTREM-1, peaking at 2 h and then progressively decreasing up to 24 h (Fig. 1H), which is consistent with the nature of this inflammatory model. While sTREM-1 appeared to be stable in human and mouse whole blood ex vivo (Fig. 1I), it showed a half-life of 6–16 min in vivo in mice (Fig. 1J). This suggests a mechanism of rapid sTREM-1 clearance in vivo, presumably via a renal route, and that the time-dependent increase in plasma sTREM-1 is due to continuous release of sTREM-1. These results show that sTREM-1 is stable in whole blood and displays a short half-life in vivo.

In contrast to the effects of low doses of LPS in vivo in humans, a 50% lethal dose of LPS in mice (intraperitoneal injection of 25 mg/kg LPS) induced an increase in plasma sTREM-1 over 24 h (Fig. 1K). In a cohort of 293 septic shock patients (Adrenoss-1, NCT02393781, see supplementary material 2 for patient characteristics), sTREM-1 was associated with survival (Fig. 1L). sTREM-1 levels were significantly higher in nonsurvivors at day 1, day 2, and day 3 following admission to the ICU (Fig. 1M), suggesting that sustained elevation of sTREM-1 levels may be associated with a poor outcome. Figure 1N indicates that survivors showed a striking reduction in the mean (±SD) delta-sTREM-1 concentration compared to nonsurvivors both at day 2 (−83.9 ± 209 pg/mL versus 45.3 ± 307.8 pg/mL, respectively; *p* = 0.0001) and at day 3 (−142.9 ± 240.8 pg/mL versus −43.9 ± 248.3 pg/mL, respectively; *p* = 0.0028). Accordingly, persistently high sTREM-1 levels during the first days following ICU admission were associated with mortality in septic shock patients. These data suggest that maintenance of high sTREM-1 levels in an acute condition is associated with a more complex outcome.

In summary, our results suggest that sTREM-1 is a marker of TREM-1 receptor activation and that maintenance of TREM-1 pathway activity over the first days following ICU admission is associated with a poor outcome. These results constitute the first rationale for using sTREM-1 as a marker of TREM-1 pathway activation and as a companion diagnostic tool for a TREM-1-targeted approach in human septic shock.

## Supplementary information

Supplementary material 1

Supplementary material 2
