# Supplementary material for: sTREM-1 is a specific biomarker of TREM-1 pathway activation
Source: Cell Mol Immunol. 2021 Jul 19;18(8):2054–6. doi: 10.1038/s41423-021-00733-5 (PMC8322270; doi:10.1038/s41423-021-00733-5)
Supplement: Supplementary file 1 — Supplementary material 1 [file 41423_2021_733_MOESM1_ESM.docx]

**Supplementary material 1**

**Methods**

**Animals**

Male (6–8 weeks) wild-type (WT) littermates and Trem-1^-/-^ C57Bl/6 mice were used. The experiments were performed in adherence to the National Institutes of Health guidelines on the use of laboratory animals and approved by our Institutional Animal Care and Use Committee.

**LPS-induced endotoxemia in mice**

Animals were randomly grouped (n=8-10) and treated with 25mg/kg *Escherichia coli* lipopolysaccharide (LPS) (0127:B8) or NaCl intraperitoneally, and animals were sacrificed at regular intervals for blood sampling (0, 2, 6 and 24hours).

For the determination of *in vivo* sTREM-1 half-life, in wild-type Trem-1^-/-^ mice or 18hours after LPS challenge, recombinant mouse TREM-1/CD354 (C-6His) protein (Novoprotein) at a dose of 2000pg/mL was administrated by orbital injection. Blood samples were collected 0.5, 1, 2, 3, 6 and 24hours following protein injection, and plasma was recovered after centrifugation of whole blood samples (300g, 10 min) and immediately stored at -80°C.

**Cell culture and stimulation**

The human myelomonocytic cell line U937 (Culture Collections, Public Health England No. 85011440, Sigma Aldrich) were seeded at 0.3x10^6^ cells/well in RPMI 1640 Glutamax supplemented with 10% fetal calf serum, 25mM Hepes, and 100 U/ml penicillin and streptomycin (all from Thermo Fisher Scientific, USA).

For the assessment of TREM-1 expression, 100nM of 1,25-dihydroxyvitamin D3 (Sigma- Aldrich, USA) was supplemented to the culture medium for 48 hours.

Depending on the experiment, U937-vitD cells were incubated under resting conditions or with different concentrations of PP (PGLYRP1 + PGN) complex during 10 minutes for immunoblotting or 24 hours for sTREM-1 and IL-6 assessments.

For confocal and shedding experiments, when indicated, cells were also incubated with LPS (*Escherichia coli* 0127:B8, 10µg/mL; Sigma-Aldrich), APMA (ρ-aminophenylmercuric acetate, 1mM; Sigma-Aldrich), pro-MMP-9 or activated-MMP9 (1µg/mL; R&D systems, Abingdon, UK). Activated MMP9 was obtained by incubating pro-MMP9 with 1mM APMA for 24hours at 37°C.

**Immunoblotting**

Total proteins were extracted from U937 stimulated cells using Phosphosafe extraction buffer (Novagen, USA), and the total protein concentration was measured by a Coomassie protein assay kit (Thermo Fisher Scientific) and used for normalization. Lysates were then analyzed by western blot (Criterion XT Bis-Tris Gel, 4–12%, Bio-Rad, USA, and polyvinylidene difluoride membrane, Millipore, France) and revealed with anti-phospho-SYK (Cell Signaling, USA) with the corresponding secondary antibody conjugated to horseradish peroxidase (GE Healthcare, USA) and Super-Signal West Femto Substrate (Thermo Fisher Scientific). Anti-SYK (Cell Signaling) were used for normalization on the same membranes after stripping. Acquisition and normalization were performed with an LAS-4000 imager (Fujifilm, Japan) and Multi-Gauge software (LifeScience Fujifilm, Japan).

***Ex vivo* LPS stimulation of human whole blood**

Blood samples collected in pyrogen free K_2_EDTA tubes from healthy volunteers were obtained from the Etablissement Français du Sang (ALC/PIL/DIR/AJR/FO/606) and used no more than 3 hours after blood withdrawal. Whole blood samples were stimulated with PBS or different doses of *Escherichia coli* lipopolysaccharide (LPS) (0127:B8) (0.05 to 100ng/mL) and incubated at 37°C under orbital agitation (450 rpm). After 1, 2, 6 and 24hours, blood was collected for further mRNA extraction, Flow Cytometry analysis, and plasma collection. Plasma was recovered after centrifugation (300g, 10 min) and immediately stored at -80°C until sTREM-1 assessments.

***Ex vivo* TREM-1 half-life assessment in whole blood**

Whole blood samples from healthy donors or Trem-1^-/-^ C57Bl/6 mice were spiked with 2000pg/mL of recombinant human TREM-1 protein (rhTREM-1, C506, Novoprotein) or mouse TREM-1 protein (rmTREM-1, CS67, Novoprotein) respectively and were incubated at 37°C under orbital agitation (450 rpm). Plasmas were recovered by centrifugation 0.5, 1, 2, 3, 6 and 24hours following rhTREM-1 spiking for further sTREM-1 quantification by ELISA.

**Confocal microscopy**

U937 cells were seeded at 0.3x10^6^ cells/well, after stimulation, cells were washed and fixed with paraformaldehyde (4%) for 20min and permeabilized with Triton 0.1 % for 30min prior to incubation with the primary antibodies (anti-hTREM-1-AF488; Bioss, USA) at 4°C overnight. Nuclei were stained with TO-PRO-3 (1µg/mL; Invitrogen, USA) for 1hour at 37 °C. After washing, coverslips were mounted using a solution of Vectashield (Vector Laboratories, USA). Confocal images were obtained using a Leica SP5 confocal laser scanning microscope system (Leica, Germany) fitted with appropriated filter sets and acquired in sequential scan mode. Images were processed using LAS-AF Lite software (Leica, Germany).

**Real-time PCR**

Total RNAs were extracted from whole blood samples using QIAamp RNA Blood Mini Kit (Qiagen, Venlo, The Netherlands) and quantified with NanoDrop (Thermo Fisher Scientific, Waltham, MA, USA) before being reverse transcribed using the iScript cDNA synthesis kit (Bio-Rad, Hercules, CA, USA) and quantified by quantitative polymerase chain reaction (PCR) using Qiagen probes (Quantitect Primers) for human TREM-1 and ActB. ActB serves as housekeeping gene.

**FACS analysis**

On U937 cells, the expression of TREM-1 was detected by flow cytometry after staining with 5µl TREM-1-APC or isotype control (Miltenyi Biotech) for 30min at 4°C in the dark. After washing with PBS twice, the cells were re-suspended and fixed in 4% paraformaldehyde solution.

In whole blood cells, 100μL of anticoagulated peripheral blood was stained with surface antibodies (hTREM-1 APC and CD14-PE, Miltenyi Biotech) for 20min at 4°C in the dark prior to red blood cells lysis and fixation with VersaLyse + 2.5% IOTest fixative solution (Beckman Coulter) for 10min in the dark. Lysed cells were washed twice with PBS prior to acquisition.

Accuri C6 flow cytometer (Becton Dickinson, San Jose, CA, USA) was used for acquisition. Flow Jow^TM^ 10 was used for analysis.

**Endotoxemia model in healthy volunteers**

sTREM-1 was quantified in plasma samples of subjects having received a 2ng/kg of body weight single injection^1^.

**Retrospective septic shock patients’ cohorts**

sTREM-1 was quantified in plasma samples of 297 septic shock patients from the AdrenOSS-1 cohort (NCT02393781^2^). Briefly, Adrenoss-1 is a European prospective, observational, multinational study in 24 ICU units from 5 countries (France, Belgium, the Netherlands, Italy, and Germany). These patients were recruited from June 2015 to May 2016. Plasma samples were collected at ICU admission, and 24hours and 48hours after admission.

**ELISA**

Murine sTREM-1 levels were assessed using enzyme-linked immunosorbent assay (Mouse/Rat TREM-1 Quantikine ELISA Kit; R&D Systems) according to the manufacturer’s protocol.

Supernatants from U937 stimulated cells were recovered after a 24-h stimulation, and IL-6 was measured using Human IL-6 Quantikine ELISA Kit (Biotechne, R&D Systems) according to the manufacturer’s instructions.

Human sTREM-1 levels were measured using an ELISA analytical method that has been validated according to regulatory requirements (EMA 2011) using an enzyme-linked immunosorbent assay (Human TREM-1 Quantikine ELISA kit, R&D Systems) The analytical performances and acceptance criteria of this method are summarized in Table 1.

**Statistical analysis**

The statistical significance of differences between two groups was analyzed using two-tailed Student’s t-test and ~~The statistical significance~~ the time-dependent differences between two groups was analyzed using two-way ANOVA test using GraphPad Prism 7.01 (GraphPad Software, USA). A p-value < 0.05 was considered significant.

In the Adrenoss cohort, the association of sTREM-1 levels at Day 1 and 28-day mortality has been investigated in a logistic regression model adjusting for sTREM-1 Day 1 levels and categorized in four classes corresponding to quartiles for the distribution of sTREM-1 at Day 1. 28-day mortality rates have been estimated within each quartile.

In-between groups (survivors versus non-survivors) and sTREM-1 changes from baseline (Day1) to Day2 and to Day3 were compared using Kruskal-Wallis test. Patients with missing values at Day 2 and Day 3 were excluded from respective analyses.

**REFRENCES**

1 van der Meer AJ, Scicluna BP, Moerland PD, Lin J, Jacobson EW, Vlasuk GP *et al.* The Selective Sirtuin 1 Activator SRT2104 Reduces Endotoxin-Induced Cytokine Release and Coagulation Activation in Humans. *Crit Care Med* 2015; **43**: e199-202.

2 Mebazaa A, Geven C, Hollinger A, Wittebole X, Chousterman BG, Blet A *et al.* Circulating adrenomedullin estimates survival and reversibility of organ failure in sepsis: the prospective observational multinational Adrenomedullin and Outcome in Sepsis and Septic Shock-1 (AdrenOSS-1) study. *Crit Care Lond Engl* 2018; **22**: 354.

| Analyte | sTREM-1 | |
| --- | --- | --- |
| Matrix | Human K_2_-EDTA plasma | |
| Analytical method | ELISA | |
| Calibration standards  (two wells per CS including anchoring points#) in Assay Diluent from the kit | 15.6#, 31.3 (LCS), 62.5, 125, 250, 500, 750, 1000, 2000 (HCS) and 3000# pg/mL  The imprecision (%CV), must be ≤20% for CS ranged from 31.3 (LCS) to 2000 pg/mL (HCS).  The inaccuracy(%RE), must be within ±20% (±25% at the LCS and the HCS).  No more than two CS (30%) may be excluded from the calibration curve, which must contain finally at least 6 calibration concentration levels, including the LCS and HCS. | |
| LLOQ in human K_2_-EDTA plasma | 34.2 pg/mL | |
| ULOQ in human K_2_-EDTA plasma | 2070 pg/mL | |
| QC samples  (n=2 duplicates, unspiked or spiked samples prepared in undiluted or diluted human K_2_-EDTA plasma) | QC.Low (93 pg/mL), QC.Mid (728 pg/mL) and QC.High (1610 pg/mL)  %CV must be ≤20% and %RE must be within ±20%.  At least 4/6 QC samples must be within acceptance criteria (2/6 QC samples, not both at the same concentration, may be outside the acceptance criteria). | |
| MRD | None | |
| Dilution linearity | Up to 1/100 in calibrator diluent RD5-18 | |
| Specificity against endogenous matrix components | No interference was demonstrated in any of the blank human K_2_-EDTA plasma samples tested. | |
| Stability in human K_2_-EDTA plasma | Freeze/thaw (F/T) stability | 3 cycles at -24°C±6°C  3 cycles at -75°C±10°C |
|  | Short-term stability | 24 hours at room temperature  24 hours at +5°C±5°C |
|  | Long-term (LT) stability | 670 days at -24°C±6°C  670 days at -75°C±10°C |

Table 1: Summary of the analytical performances and acceptance criteria of the sTREM-1 ELISA validated method

LCS: lowest calibration standard; HCS: highest calibration standard; CS: calibration standard(s); QC: quality control; RE: relative error; MRD: minimum required dilution; LLOQ: lower limit of quantification; ULOQ: upper limit of quantification
