# Supplementary material for: sTREM-1 is a specific biomarker of TREM-1 pathway activation
Source: Cell Mol Immunol. 2021 Jul 19;18(8):2054–6. doi: 10.1038/s41423-021-00733-5 (PMC8322270; doi:10.1038/s41423-021-00733-5)
Supplement: Supplementary file 2 — Supplementary material 2 [file 41423_2021_733_MOESM2_ESM.docx]

|  |  | **sTREM-1 Q1** | **sTREM-1 Q2** | **sTREM-1 Q3** | **sTREM-1 Q4** |  |
| --- | --- | --- | --- | --- | --- | --- |
|  | **all** | **(42,299]** | **(299,497]** | **(497,809]** | **(809,5.54e+03]** | **p-value** |
| **Characteristics** | **n=293** | **n=74** | **n=73** | **n=73** | **n=73** |  |
| sTREM-1 at admission (pg/ml) | 497 [299-809] |  |  |  |  |  |
| Age (year) | 67.2 [57.8-77.1] | 63.2 [52.5-70.0] | 69.8 [60.0-76.4] | 69.9 [58.7-81.1] | 66.7 [60.4-77.1] | 0.0055 |
| Females (No. %) | 106 (36.2) | 22 (29.7) | 27 (37) | 28 (38.4) | 29 (39.7) | 0.5931 |
| Body Mass Index (kg/m2) | 25.9 [23.0-30.4] | 25.9 [22.9-29.6] | 25.4 [22.5-29.0] | 26.7 [23.3-30.0] | 28.4 [23.9-33.3] | 0.1056 |
| **Type of ICU admission:** |  |  |  |  |  | 0.1452 |
| Medical | 215 (73.4) | 55 (74.3) | 53 (72.6) | 54 (74) | 53 (72.6) |  |
| Surgical - emergency procedure | 67 (22.9) | 19 (25.7) | 14 (19.2) | 15 (20.5) | 19 (26) |  |
| Surgical - elective procedure | 11 (3.8) | 0 (0) | 6 (8.2) | 4 (5.5) | 1 (1.4) |  |
| **Origin of sepsis:** |  |  |  |  |  | 0.4141 |
| Lung | 81 (27.6) | 26 (35.1) | 24 (32.9) | 16 (21.9) | 15 (20.5) |  |
| Blood stream | 40 (13.7) | 5 (6.8) | 8 (11) | 14 (19.2) | 13 (17.8) |  |
| Urinary tract | 50 (17.1) | 9 (12.2) | 13 (17.8) | 14 (19.2) | 14 (19.2) |  |
| Catheter | 19 (6.5) | 4 (5.4) | 4 (5.5) | 8 (11) | 3 (4.1) |  |
| Peritonitis | 17 (5.8) | 4 (5.4) | 3 (4.1) | 6 (8.2) | 4 (5.5) |  |
| Endocarditis | 18 (6.1) | 6 (8.1) | 4 (5.5) | 3 (4.1) | 5 (6.8) |  |
| Bile duct infection | 4 (1.4) | 1 (1.4) | 1 (1.4) | 2 (2.7) | 0 (0) |  |
| CNS | 3 (1) | 2 (2.7) | 0 (0) | 0 (0) | 1 (1.4) |  |
| Other | 61 (20.8) | 17 (23) | 16 (21.9) | 10 (13.7) | 18 (24.7) |  |
| **Medical history OR specific diseases (COPD, DMII, immunodysfunction, chronic renal failure, ischemic or congestive heart disease)** | | | | | | |
| any cardiac comorbidity (yes) | 213 (72.7) | 47 (63.5) | 49 (67.1) | 59 (80.8) | 58 (79.5) | 0.0386 |
| any non-cardiac comorbidity (yes) | 226 (77.1) | 56 (75.7) | 59 (80.8) | 56 (76.7) | 55 (75.3) | 0.8513 |
| any chronicmediaction (yes) | 204 (69.6) | 51 (68.9) | 53 (72.6) | 52 (71.2) | 48 (65.8) | 0.8182 |

Supplementary table 1: Adrenoss cohort patients characteristics
